# An Improved Quantitative Real-Time PCR Assay for the Enumeration of *Heterosigma akashiwo* (Raphidophyceae) Cysts Using a DNA Debris Removal Method and a Cyst-Based Standard Curve

**DOI:** 10.1371/journal.pone.0145712

**Published:** 2016-01-07

**Authors:** Joo-Hwan Kim, Jin Ho Kim, Pengbin Wang, Bum Soo Park, Myung-Soo Han

**Affiliations:** 1 Department of Life Science, Hanyang University, Seoul 133–791, South Korea; 2 Research Institute for Natural Sciences, Hanyang University, Seoul 133–791, South Korea; University of Connecticut, UNITED STATES

## Abstract

The identification and quantification of *Heterosigma akashiwo* cysts in sediments by light microscopy can be difficult due to the small size and morphology of the cysts, which are often indistinguishable from those of other types of algae. Quantitative real-time PCR (qPCR) based assays represent a potentially efficient method for quantifying the abundance of *H*. *akashiwo* cysts, although standard curves must be based on cyst DNA rather than on vegetative cell DNA due to differences in gene copy number and DNA extraction yield between these two cell types. Furthermore, qPCR on sediment samples can be complicated by the presence of extracellular DNA debris. To solve these problems, we constructed a cyst-based standard curve and developed a simple method for removing DNA debris from sediment samples. This cyst-based standard curve was compared with a standard curve based on vegetative cells, as vegetative cells may have twice the gene copy number of cysts. To remove DNA debris from the sediment, we developed a simple method involving dilution with distilled water and heating at 75°C. A total of 18 sediment samples were used to evaluate this method. Cyst abundance determined using the qPCR assay without DNA debris removal yielded results up to 51-fold greater than with direct counting. By contrast, a highly significant correlation was observed between cyst abundance determined by direct counting and the qPCR assay in conjunction with DNA debris removal (*r*^2^ = 0.72, slope = 1.07, *p* < 0.001). Therefore, this improved qPCR method should be a powerful tool for the accurate quantification of *H*. *akashiwo* cysts in sediment samples.

## Introduction

*Heterosigma akashiwo* is a HAB-inducing resident of many coastal environments and is recognized as a toxic, fish-killing phytoplankton. This species has significant negative impacts on fisheries that can cost the aquaculture industry millions of dollars each year [1–4]. *H*. *akashiwo* is known to have three life stages: vegetative cells, resting cells, and cysts [5–7]. Vegetative cells are generally heart-shaped, although they can be quite variable and irregular, whereas resting cells and cysts are nearly spherical. Both vegetative cells and resting cells have two flagella, but the motility of resting cells is either non-existent or extremely low. By contrast, cysts have a ridged cell wall and no flagella. Although both vegetative cells and cysts have been found *in situ*, resting cells have only been observed under unfavorable *in vitro* condition [7, 8]. *H*. *akashiwo* cysts are known to play an important role in bloom initiation [8, 9]. To more accurately evaluate the role of cysts in the bloom mechanisms of *H*. *akashiwo*, we must develop a sensitive and accurate method for quantifying cyst numbers. Due to the small size and cryptic morphology of *H*. *akashiwo* cysts, they are most often quantified using indirect means, such as the most probable number (MPN) method, rather than direct counting with light microscopy [6, 8–11]. However, the MPN method has the potential to both under- and overestimate sediment cyst abundance [12, 13]. In addition, the MPN method is not appropriate for large-scale sampling (i.e., many sample stations over long time scales) due to the time-consuming and laborious processes required for pre-treatment and observation [14]. Therefore, alternative techniques must be developed to efficiently and accurately quantify cyst abundance in sediment samples.

Quantitative real-time PCR (qPCR) is widely known as a sensitive, accurate, and efficient technique for quantifying phytoplankton in the vegetative stage [15–17]. However, qPCR assays for quantifying phytoplankton in the resting stage have not been as well developed and are often inaccurate [18–20]. In particular, the difference in rRNA gene copy number between cysts and vegetative cells can induce accuracy errors [19, 21, 22]. Also, unlike vegetative cells, algal cysts have thick cell walls, which can lead to relatively low DNA extraction yields compared with vegetative cells, a potential source of error when performing qPCR assays [19]. Hence, the construction of qPCR standard curves based on cysts rather than vegetative cells should be a priority. Finally, a significant hurdle to previous qPCR-based studies involving cyst quantification was the presence of large amounts of extracellular DNA in the sediment [23–26]. This extracellular DNA debris, which can include target species DNA, can lead to considerable overestimation when using qPCR-based assays [20]. Therefore, a method for removing extracellular DNA debris is highly necessary to accurate quantification for resting cysts in sediment.

To quantify *H*. *akashiwo* cyst abundance, Portune *et al*. [18] developed and applied qPCR assays on field samples. However, they did not consider the difference in rRNA gene copy number between actual cysts and vegetative cells. In addition, DNA debris present in the sediments, which can significantly affect qPCR accuracy, was not also considered, similar to previous qPCR-based cyst quantification studies [19, 21, 22].

In this study, to improve qPCR for cyst quantification, we constructed standard curve using isolated *H*. *akashiwo* cysts from natural sediments and developed a method to remove DNA debris from the sediment, which heretofore had not been addressed in studies monitoring harmful algal cysts.

## Materials and Methods

### Ethics Statement

No specific permits were required for the sampling, as the location (Youngsan River estuarine bay: 34°47’N, 126°25’E) was not privately-owned or protected and the field studies did not involve endangered or protected species.

### Collection and pre-treatment of sediment samples

**Fig 1:**
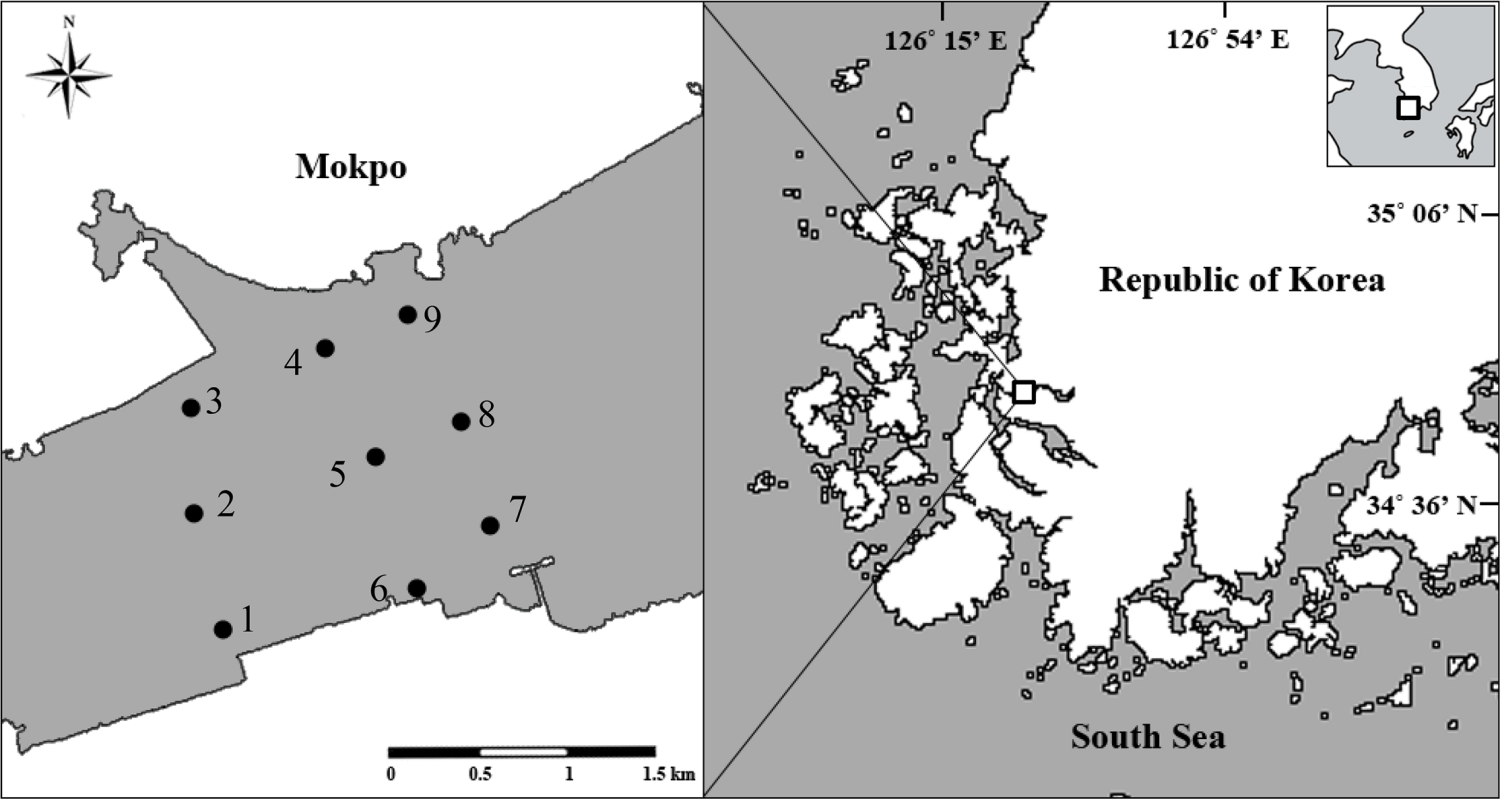
Sampling location at the Youngsan River estuarine bay, Republic of Korea.

Sediment samples were collected from the Youngsan River estuarine bay located on the southwest coast of Korea during November 2012 (Fig 1). Dense blooms of *H*. *akashiwo* often occur in this highly eutrophic region. Duplicate sediment samples were collected using a gravity core (2 cm diameter) at nine stations and transported to the laboratory on ice under dark conditions. Next, 1 cm of the top sediment layer was transferred to a dark plastic bottle (50 mL). To produce a sediment suspension, 1 g (wet weight) of well-mixed sediment was transferred to a 15 mL conical tube (SPL, Korea) and gently resuspended in 10 mL cool, filtered seawater (grade GF/F with a nominal pore size of 0.7 μm; Whatman, USA). Suspensions were stored in the dark at 4°C until the samples were ready for direct counting and qPCR analysis.

### Isolation of *Heterosigma akashiwo* cysts

To isolate natural *H*. *akashiwo* cysts, sediment suspensions were sonicated three times for 10 sec (UT 53N, Sharp, Japan) to break up sediment aggregates and to separate cysts from the sediment particles [6]. A 200 μL aliquot of the sediment suspension was diluted with 6 mL filtered seawater and spread on a Petri dish (60 × 15 mm; SPL, Korea). *H*. *akashiwo* cysts were isolated using a capillary pipette under an epifluorescence microscope (IX71, Olympus, Japan) equipped with a chlorophyll filter set (450–490 nm band-pass excitation, 520 nm long-pass emission). *H*. *akashiwo* cyst identification was conducted following the morphological description of Imai *et al*. and Kim *et al*. [6, 27]. For DNA extraction, isolated cysts were transferred to a microfuge tube containing beads and 750 μL lysis buffer from the PowerSoil^®^ DNA Isolation Kit (MoBio, USA) and maintained at 4°C in the dark until DNA extraction. DNA from these extractions was used to construct the qPCR standard curves.

### DNA extraction from cysts and vegetative cells for qPCR standard curve construction

To construct standard curves based on *H*. *akashiwo* cysts, we used DNA extracted from cells. According to Hou *et al*. [28], qPCR assays based on circular plasmid standards can lead to significant overestimation of DNA levels. To increase the accuracy of our quantification method, we therefore chose to use a standard based on cellular DNA. DNA was extracted from 213 isolated cysts using the PowerSoil® DNA Isolation Kit, according to the manufacturer’s protocol. This kit contains a bead beating step that breaks open cysts as well as extraction steps that remove PCR inhibitors from the sediment. According to Ning *et al*., this kit gives a higher DNA yield than the UltraClean Soil DNA kit (MoBio) [29], which was previously used to detect *Pfiesteria* spp. and *Cochlodinium* spp. cells in sediment [19, 20, 30]. To extract DNA from vegetative cells, *H*. *akashiwo* (HYM06HA) was grown at 20°C in 50 mL culture flasks (SPL, Korea) containing 20 mL f/2 medium [31] under cool-white fluorescent lamps (100 μE m^-1^s^-1^ photon flux) under a 12:12 h light:dark photoperiod. *H*. *akashiwo* vegetative cells were counted during the exponential growth phase using a hemocytometer (Marienfeld, Germany) under a light microscope at 200× magnification. Approximately 8,000 cells were transferred to a microfuge tube containing beads and the lysis buffer from the PowerSoil® DNA Isolation Kit. Sampling was performed 3 h after the lights were turned on in order to only obtain G1 phase cells [32]. DNA extraction of the vegetative cells was performed using the same process as for the cysts and then diluted 40-fold in TE buffer (10 mM Tris-HCl, 1.0 mM EDTA, pH 8) to match the concentration of the cyst DNA for standard curves construction.

### Method for the removal of DNA debris in the sediment

Cyst quantification results based on qPCR assays can be skewed due to DNA from dead vegetative cells present in the sediment [23]. Therefore, the removal of DNA debris from the sediment is necessary for the accurate quantification of *H*. *akashiwo* cysts by qPCR. To solve this problem, sediment samples were subjected to various temperature and ion concentration treatment conditions to determine the optimum conditions for the removal of DNA debris.

**Fig 2:**
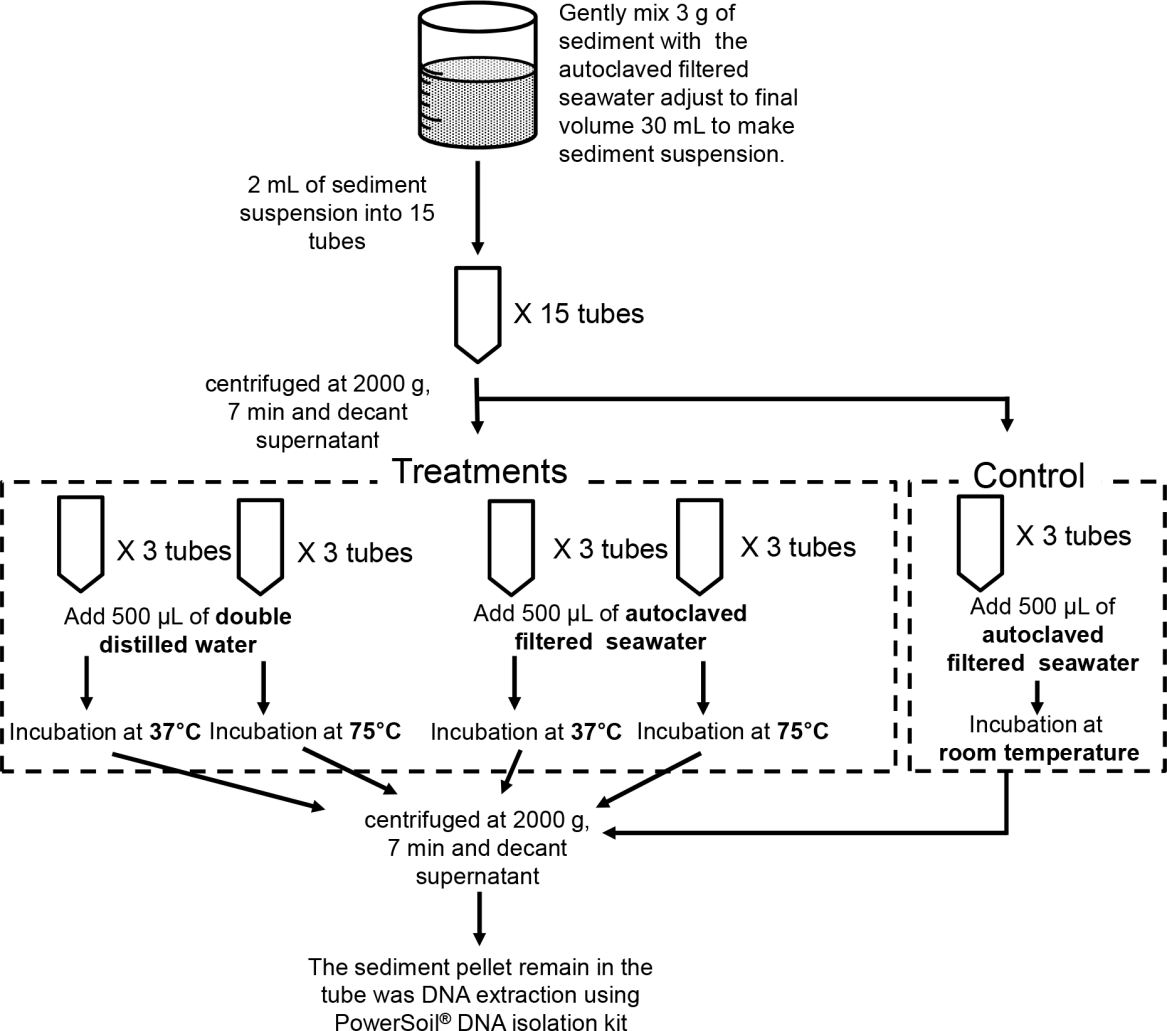
Schematic of the DNA debris removal process for the sediment samples.

Four experimental treatments and one control were examined (Fig 2). A 30 mL sediment suspension containing 3 g sediment was prepared. Two milliliter aliquots of the suspension were transferred to fifteen 2 mL microfuge tubes and then centrifuged (2,000 g for 7 min). The pellets were resuspended by vortexing in 500 μL autoclaved filtered seawater (SW) or distilled water (DW) to dilute the saline. The suspensions were incubated at 37°C or at 75°C in a water bath for 10 min. For the control sample, 2 mL sediment suspension was transferred to three 2 mL microfuge tubes and centrifuged (2,000 g for 7 min). After decanting the supernatants, the pellets were re-suspended in 500 μL autoclaved filtered SW and incubated at room temperature for 10 min. All treatment and control samples were centrifuged (2,000 g for 7 min), after which the supernatant was carefully removed. The sediment pellets were mixed with beads and a lysis buffer from the PowerSoil® DNA Isolation Kit (MoBio), and DNA extraction was conducted according to the manufacturer’s instructions. All of the DNA samples, including the experimental treatments and control, were prepared in triplicate and were analyzed using the *H*. *akashiwo*-specific qPCR assay described below.

To determine whether heat and/or osmotic pressure could damage the cysts, we observed *H*. *akashiwo* cysts after treatment. First, *H*. *akashiwo* cysts were placed in 96-well plates (Falcon, USA) that were filled with either DW or autoclaved filtered SW. The plate was floated in a 75°C water bath for 10 min, and the cysts were then observed using an inverted microscope to determine whether the cysts were destroyed. Then, the cysts were fixed with glutaraldehyde (final concentration 1%) and stained with 4’-6-diamidino-2-phenylindole (DAPI) (0.5 μg mL^-1^). To observe the cyst nuclei, stained cysts were observed under an epifluorescence microscope (BX-50, Olympus, Japan) equipped with a UV filter with an excitation range of 330–385 nm and emission range of >420 nm at 1000× magnification.

To evaluate whether our DNA debris removal method could be applied to other types of algae, we also tested a variety of other algal cysts. In total, 33 different cysts were isolated from sediment sampled from Tongyeong, Korea (34°46'39" N, 128°23'06" E). Each cyst was photographed on a glass slide using an inverted microscope (IX71, Olympus, Japan), and then the cysts were placed in 96-well plates filled with DW. The plate was floated in a 75°C water bath for 10 min, and the cysts were then isolated and photographed on glass slides using an inverted microscope to confirm whether the cysts were destroyed.

### Construction of standard curves dependent on different life stages

Serial 2-fold dilutions (1- to 16-fold) of DNA samples from both cysts and vegetative cells were used to construct the standard curves using triplicate measurements from real-time PCR. The sequences of the *H*. *akashiwo-*specific PCR primers are shown in Table 1. This primer set targets the species-specific internal transcribed spacer 2 region located between the 5.8S and 28S rRNA genes. This primer pair is specific to *H*. *akashiwo* and does not react to other algal species [33].

**Table 1:** Sequence information for the *Heterosigma akashiwo*-specific primer set.

Target species	Primer	Sequence (5'-3')	Target sequence length	Optimal annealing temperature	Reference
	HASF2 (forward)	GTT GGA CCA TCA CCC CCT TTT			
***Heterosigma akashiwo***			148 bp	62.5°C	Park *et al*. [33]
	HASR2 (reverse)	GGA GGA TGT GTG ATC GTG AAG AT			

The EvaGreen Real-Time PCR assays contained 7.5 μL 1× SsoFast™ EvaGreen^®^ Supermix (Bio-Rad, CA, USA), 0.25 μM primer set, 2 μL genomic DNA (~0.1 μg), and double-distilled water in a final volume of 15 μL. The qPCR reactions were performed using a Chromo4 Detection System (Bio-Rad, USA) at 98°C for 3 min, followed by 39 cycles at 98°C for 7 s and 62.5°C for 10 s. After denaturation, the melt curves were monitored from 65°C to 95°C in 0.5°C increments using a 10 s hold at each step. All sediment DNA samples were analyzed by qPCR, and standard curves were generated using the same PCR conditions as described above.

The slope of standard curves were used to calculate PCR efficiency using the following equation: *E* = 10^(-1/*s*)^-1, where *E* is the PCR efficiency, *s* is the slope of standard curves.

### Field application of the DNA debris removal method and qPCR assay for the quantification of *Heterosigma akashiwo* cysts

DNA was extracted from 18 samples collected from nine stations situated along the Youngsan River estuarine bay after treatment (incubation in DW at 75°C), as described above, and compared with the control (incubation in autoclaved filtered SW at room temperature). To increase PCR efficiency, extracted DNA samples were diluted from 1- to 40-fold with TE buffer, depending on the initial DNA concentration (treatment group: 1- to 5-fold; control group: 20- to 40-fold), to fall within the range of the standard curve.

To compare the results of the qPCR assays, *H*. *akashiwo* cysts were counted using an epifluorescence microscope (IX71, Olympus, Japan) equipped with a chlorophyll filter (450–490 nm band-pass excitation, 520 nm long-pass emission). In particular, a 200 μL sediment suspension was diluted with 6 mL filtered SW in a Petri dish (60 × 15 mm; SPL, Korea), and direct counting was performed 5 times per sample using the same sediment suspensions conditions used for the DNA extraction.

### Statistical analysis

Statistical analyses were conducted using one-way analysis of variance (ANOVA) to compare the results among four DNA debris removal treatment groups (37°C SW, 75°C SW, 37°C DW, 75°C DW), the control group, and the direct counting groups using the SPSS version 8.0 software program (IBM, USA).

## Results

### Construction of qPCR standard curves based on DNA from cysts and vegetative cells

**Fig 3:**
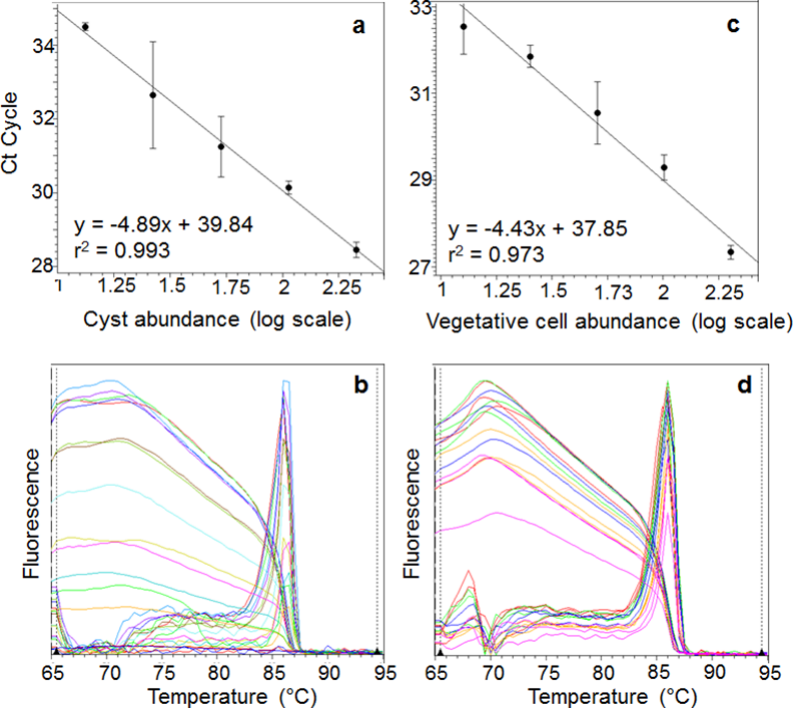
qPCR standard curves and melt curves. Standard curves were generated from triplicate serial dilutions of DNA from *H*. *akashiwo* cysts and vegetative cells (2-fold). *H*. *akashiwo* cysts (a) and *H*. *akashiwo* vegetative cells (c). Melt curves from the EvaGreen-based assays with serial dilutions of *H*. *akashiwo* cyst and vegetative cell DNA extracts: *H*. *akashiwo* cysts (b) and *H*. *akashiwo* vegetative cells (d). The melting temperature was 86°C and the peak width was 1.82.

A series of 2-fold serial dilutions was prepared from the cyst DNA extracts (213 to 13 cysts) to construct a standard curve (Fig 3A). A strong correlation was observed between the Ct values and the log of the number of cysts present in the reaction mix (*r*^2^ ≥ 0.993; slope of the correlation straight line, −4.89), with a PCR efficiency of 60% (Fig 3A). The lower cyst detection limit was 8 cysts per reaction within the linear range of cyst numbers. Standard curves based on vegetative cells were constructed to compare the standard curves of the *H*. *akashiwo* cysts and vegetative cells. A series of 2-fold serial dilutions (DNA from 202 to 12 vegetative cells) was prepared. The correlation between the Ct values and the log of the number of vegetative cells showed a linear relationship (*r*^2^ ≥ 0.973; slope of the correlation straight line, −4.43), with a PCR efficiency of 68% (Fig 3C). Melting curve analysis and sequencing of the PCR products confirmed that these PCR assays only amplified the target *H*. *akashiwo* DNA (Fig 3B and 3D).

### Evaluation of DNA debris removal methods

**Fig 4:**
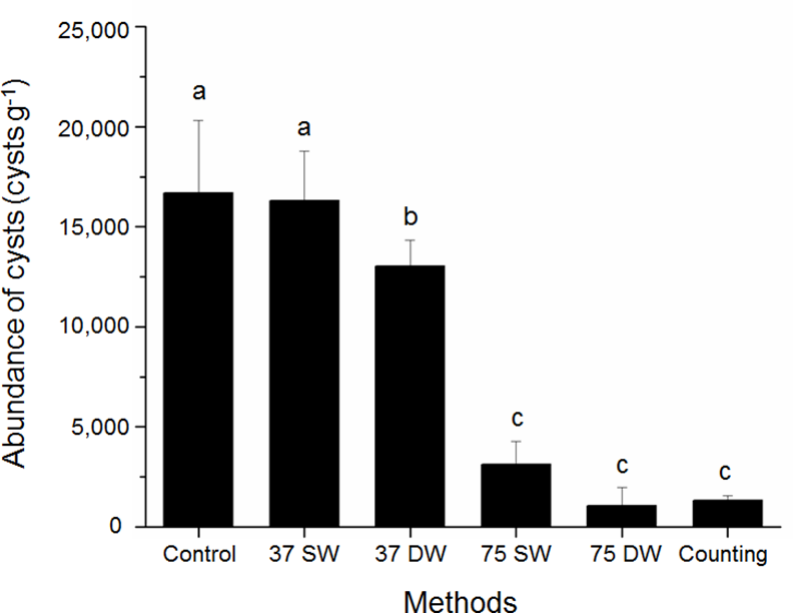
Abundance of *H*. *akashiwo* cysts calculated by qPCR after treatment with different conditions. Sediment samples were analyzed using qPCR assay after treatment under four conditions for DNA debris removal. The results from the qPCR assay were compared with results from the direct counting method. 'SW' and 'DW' samples were treated in autoclaved filtered seawater and distilled water, respectively. '37' and '75' samples were treated at 37°C and 75°C, respectively. Columns labeled with the different lower case letters within each respective treatment for each experiment were significantly different (*p* < 0.05).

To evaluate the different methods designed to remove residual DNA from the sediment, all sediment samples were treated with the removal methods described in the Materials & Methods section and analyzed by qPCR to determine the number of *H*. *akashiwo* cysts in the sample. For the control, in which the samples were incubated at room temperature in filtered SW, we calculated a cyst density of 1.66 × 10^4^ cysts g^-1^. This result was approximately 12-fold higher than the actual cyst number determined using the direct counting method. For method 1, in which the samples were incubated at 37°C in filtered SW (37°C SW), we calculated a cyst density of 1.63 × 10^4^ cysts g^-1^, similar to that of the control (Fig 4). For method 2, in which the samples were incubated at 37°C in DW (37°C DW), we calculated a cyst density of 1.30 × 10^4^ cysts g^-1^, which was slightly lower than in the control sample and in the sample incubated at 37°C in SW. For methods 3 and 4, in which samples were incubated at 75°C in filtered SW (75°C SW) (0.31 × 10^4^ cysts g^-1^) or DW (75°C DW) (0.10 × 10^4^ cysts g^-1^), respectively, we observed cyst counts that were much closer to the actual cyst number.

ANOVA revealed significant differences among the groups. The mean value for the 37°C SW method was 1.63 × 10^4^ cysts g^-1^, which was not significantly different from the control (1.66 × 10^4^ cysts g^-1^). The mean value for the 37°C DW method (1.30 × 10^4^ cysts g^-1^) was lower than that of the control, although not significant. The mean values for the 75°C SW (1.0 × 10^3^ cysts g^-1^) and 75°C DW (3.1 × 10^3^ cysts g^-1^) methods were significantly lower than that of the control (*p* < 0.05). Although the results for these last two groups were not statistically different, the 75°C DW method resulted in a lower cyst density than the 75°C SW method and yielded counts that were most similar to the direct counting method (Fig 4). Therefore, we concluded that the 75°C DW treatment was the optimal method for removing DNA debris from sediment.

**Fig 5:**
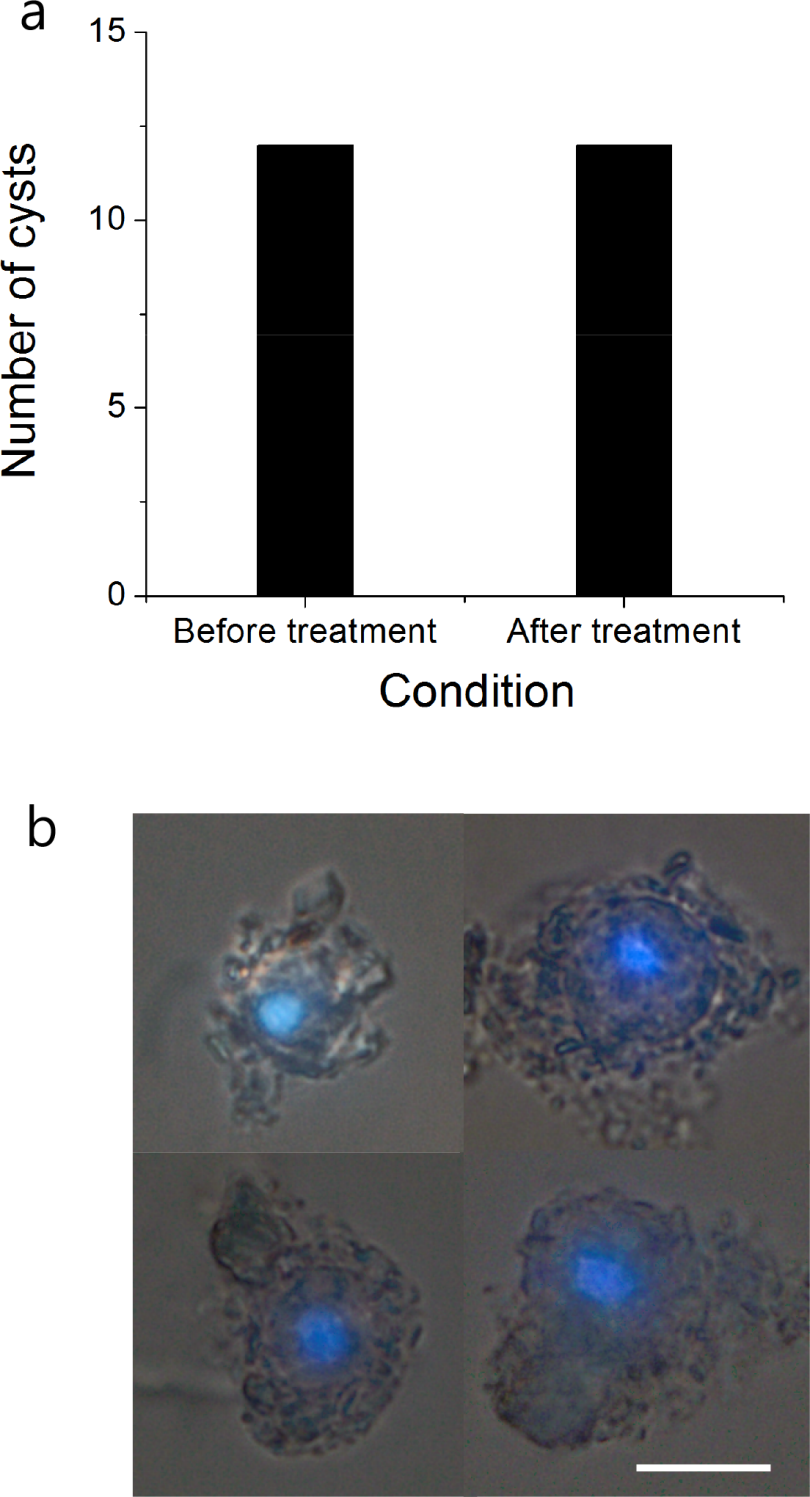
The effects of DNA debris removal process on *H*. *akashiwo* cysts. Changes in number of normal cysts (i.e., not destroyed) before and after treatment in distilled water at 75°C (a). Nuclei of *H*. *akashiwo* cysts after treatment in distilled water at 75°C and stained with DAPI (b). Scale bar = 10 μm.

We also confirmed that *H*. *akashiwo* cysts treated with this DNA debris removal method were not destroyed by the treatment and indeed were well preserved (Fig 5). Although the cysts treated with heat and DW exhibited slight cellular disorganization, the nuclei remained intact and were readily observable by epifluorescence microscopy with DAPI staining (0.5 μg/mL).

**Fig 6:**
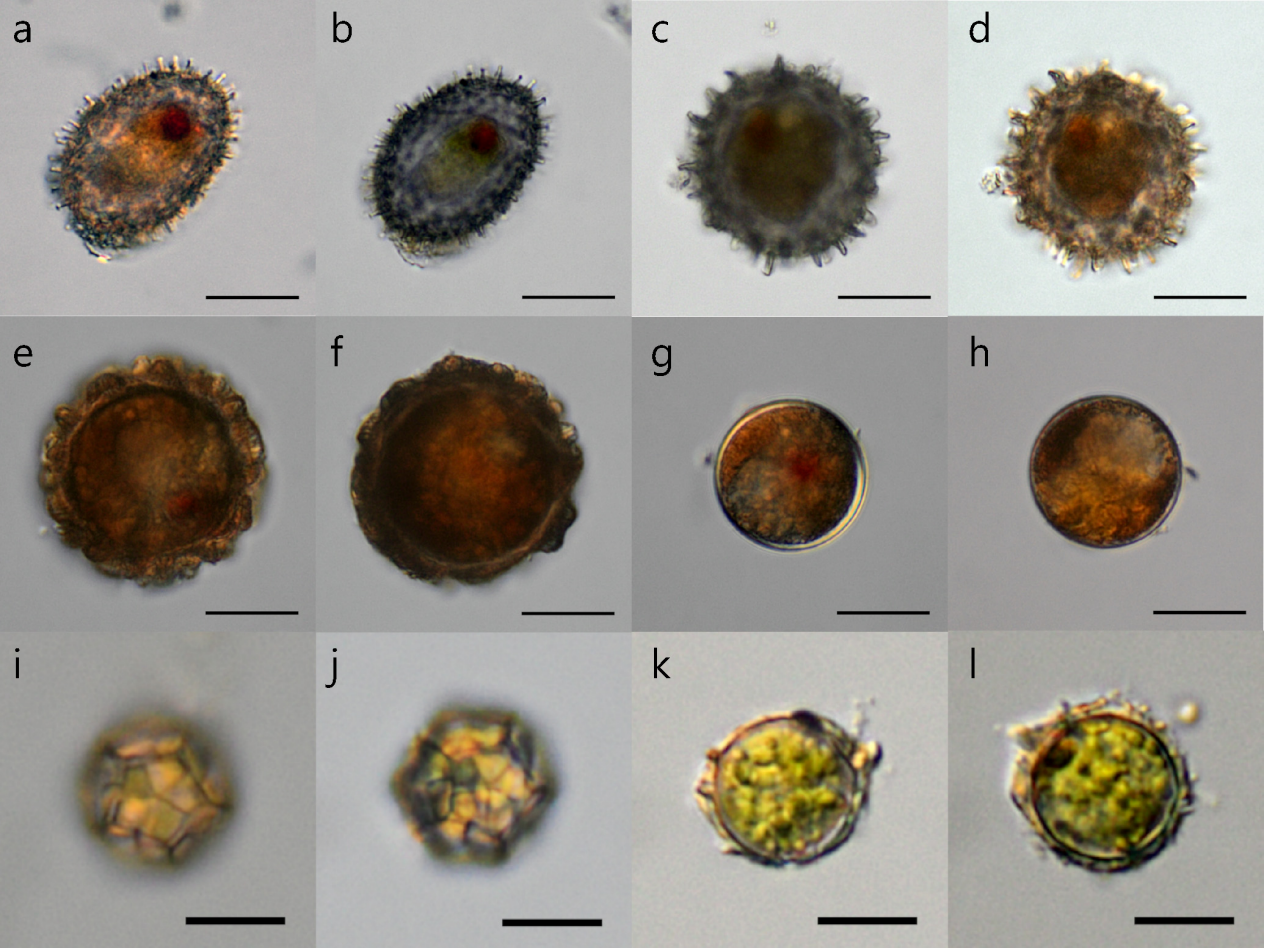
The effects of DNA debris removal process on various algal cysts. Before (a, c, e, g, i, k) and after (b, d, f, h, j, l) treatment with the optimal DNA debris removal method (75°C DW) for a variety of cysts of different types and sizes isolated from the sediment. Scale bars in (a-h) are 20 μm, scale bars in (i-l) are 10 μm.

To evaluate the applicability of this DNA removal method for other algal cysts, we isolated various cysts and exposed them to the 75°C DW method (Fig 6). Across a range of cyst types and sizes, none of the 33 different cysts we tested were destroyed by this method, and their cellular contents were generally well preserved.

### Field application of the DNA debris removal method compared with direct counting

**Fig 7:**
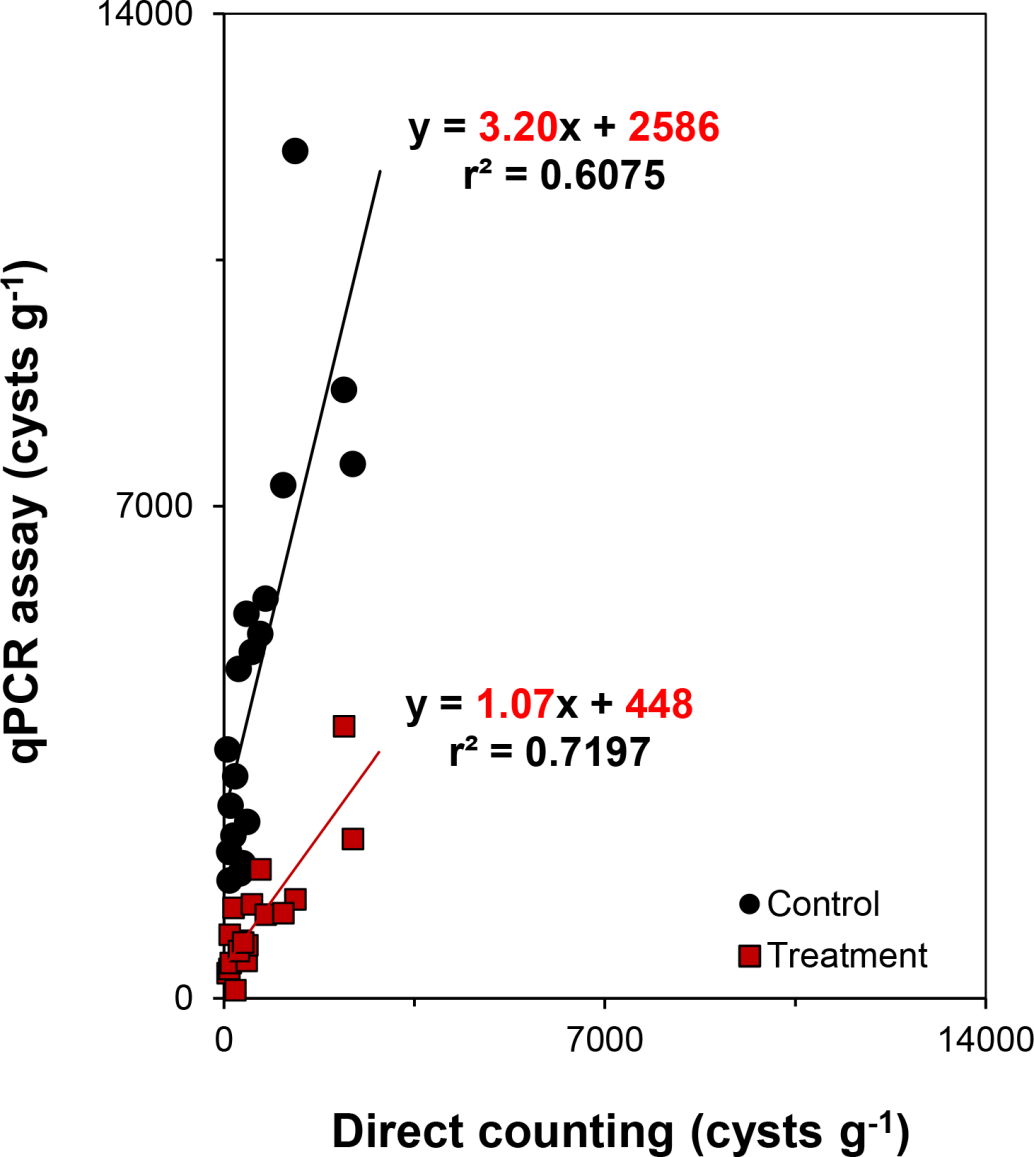
The changes of correlation between two results through direct counting method and qPCR with/without DNA debris removal treatment. The *black circle* indicates the correlation between the qPCR assay from the control samples and the direct counting method. The *red square* indicates the correlation between the qPCR assay from samples in which DNA debris was removed and the direct counting method. The *solid line* and equation represent the best linear curve fit to the data. The cyst values calculated from the control and treatment methods were significantly correlated with the direct counting values (*p* < 0.001).

After confirming that the cysts were not destroyed by the treatment, we analyzed 18 field-collected samples using the qPCR assay in conjunction with the 75°C DW DNA debris removal method (treatment). These results were compared with samples that were not treated with the DNA debris removal step (control) and with the direct counting results (Fig 7). The control and direct counting methods showed a statistically significant relationship with abundance (*r*^2^ = 0.60, slope = 3.20, *p* < 0.001). However, the control abundance was, on average, approximately 13-fold higher than the actual cyst abundance determined by direct counting (3.2- to 51.7-fold). By contrast, the 75°C DW treatment group showed a significant relationship with the direct counting method and was much more reliable than the controls, avoiding the problem of overestimation (*r*^2^ = 0.72, slope = 1.07, *p* < 0.001) (Fig 7).

## Discussion

It is generally recognized that the enumeration of *H*. *akashiwo* cysts by light microscopy is inherently inaccurate, due to the extremely small size and cryptic morphology of the cysts [6, 8, 10, 11, 34]. To improve the accuracy of quantification, researchers have attempted to develop qPCR-based assays for counting cysts [18]. One of most crucial aspects of qPCR assays is the construction of standard curves, as these will directly affect the final calculated values. There have been many studies on the differences in rRNA gene copy number between vegetative cells and cysts, which are based on ploidy states, and on whether these copy number differences are affected by environmental conditions [18, 19, 35]. Therefore, qPCR assays for quantifying cysts have been applied based on the rRNA gene copy number of the cysts [19, 21, 22]. In the case of *H*. *akashiwo*, it has been suggested that the DNA content of cysts is lower than that of vegetative cells because the nucleus is smaller in cysts [6, 36]. Nevertheless, in a previous study, a standard curve was constructed based on the rRNA gene copy number of the vegetative cells and not the actual cysts [18].

In the present study, we first attempted to collect *H*. *akashiwo* cysts from natural sediments and construct a qPCR standard curve based on isolated cysts. The standard curves based on cyst number were also significant (*r*^2^ > 0.993) (Fig 3A) and melt curve analysis confirmed that all the melt curves had one informative peak, indicating that only the target region was successfully amplified (Fig 3B).

Interestingly, as shown in Table 2, there were differences between the quantification results from the two standard curves based on DNA from cysts or vegetative cells. On average, the cyst abundance data using the vegetative cell-based standard curves were approximately 2-fold lower than from the cyst-based standard curves, suggesting that the rRNA gene copy number of cysts is lower than that of vegetative cells (Table 2). However, there are two possible explanations for this difference in cyst abundance. The first is that DNA extraction yields are different between vegetative cells and cysts. Indeed, the presence of the cyst cell wall is an important factor that can affect DNA yield, and ultimately, cyst quantification [19]. In the present study, the cell wall of *H*. *akashiwo* cysts was shown to be durable enough to endure high-temperature conditions, which could potentially hamper the DNA extraction process (Fig 5). Therefore, the DNA yield from cysts might be lower than from vegetative cells, resulting in the appearance of a lower rRNA gene copy number in cysts compared with vegetative cells. Alternatively, it is possible that the ploidy level of the cysts is actually lower than that of the vegetative cells. It has been reported that both *Chattonella marina* var. *maria* and *C*. *marina* var. *antiqua* (Raphidophyceae) cysts are haploid, whereas their vegetative cells are diploid [36]. A single study suggested that *H*. *akashiwo* vegetative cells and resting cell have the same ploidy level [7]. However, true cysts can be distinguished from resting cells based on obvious morphological characteristics, and resting cells have not been reported in natural sediments. In this study, we did not directly measure the DNA content of the cysts and vegetative cells of *H*. *akashiwo*. Therefore, to more accurately quantify cyst abundance, further studies on rRNA gene copy number differences between cysts and vegetative cells will be required. In particular, if the rRNA gene copy number between cysts and vegetative cells is found to be the same, then the simpler technique of using vegetative cells to generate standard curves would be appropriate.

**Table 2:** Comparison between *Heterosigma akashiwo* cyst quantification using vegetative cell- and cyst-based standard curves.

Sediment samples	Vegetative cell^a^	Cyst^a^	V/C^b^
**1**	147 ± 31	361 ± 91	0.41
**2**	311 ± 66	507 ± 49	0.61
**3**	561 ± 131	825 ± 155	0.68
**4**	1524 ± 0	3876 ± 467	0.39
**5**	244 ± 12	913 ± 33	0.27
**6**	358 ± 79	1414 ± 411	0.25
**Average**			0.44

^a^Cyst abundance calculated using either the vegetative cell- or cyst-based standard curves. Each value represents cysts per gram of sediment (wet weight).

^b^Vegetative cell:cyst ratio.

In this study, the efficiency of PCR reaction was relatively low than 70% in both standard curves of cysts and vegetative cells. Also, *r*^*2*^ values were under 0.995 which indicated variations in the data set. Generally, PCR efficiency based on the slope of the standard curves should be 90–110% in qPCR study. According to Zimmermann *et al*. [37], low concentration of DNA templates could lead to low PCR efficiency. Unfortunately, the maximum concentration of DNA template used for standard curves corresponded to only 213 cysts due to the difficulty of cyst isolation in sediment sample. Also, we adjusted maximum concentration of DNA template used in standard curves of vegetative cells to 202 cells that similar number of cysts used standard curves to compare quantification data obtained from each standard curves (Table 2). According to Park *et al*. [33], they successfully applied qPCR assay for quantification of *H*. *akashiwo* vegetative cells using same primer and PCR condition in this study. The PCR efficiency of their study was 100%, the maximum cell concentration for standard curves was above 10^5^. In this study, low DNA concentration of standard curves might cause low PCR efficiency and *r*^*2*^ value. In study of Kamikawa *et al*. [21], they constructed cyst based standard curves of *Alexandrium tamarens*e, the number of cysts for standard curves was 3,000. It was possible to collecting high number of cysts due to *Alexandrium* could be induced to form cysts in laboratory condition. However, real cyst of *H*. *akashiwo* could not be induced in current laboratory condition. Thus, further study will be needed to construct more reliable standard curves based on cysts through cysts inducing in laboratory.

Another crucial factor affecting qPCR accuracy is the presence of DNA debris in the sediment. DNA debris can be preserved for long periods in marine sediment particles and sand [23, 38]. DNA debris can introduce serious quantification errors when using qPCR-based assays, as these rely on DNA content to infer cell number; or in other words, the number of cysts in a sample can be overestimated due to the presence of residual target species DNA debris. In this study, we assumed that there would be a large amount of extracellular *H*. *akashiwo* DNA debris in the sediment samples from the Youngsan River estuarine bay. Indeed, the qPCR results showed very high cyst abundance, which was at odds with the microscopic data (Fig 7). Moreover, based on data from the National Fisheries Research and Development Institute in Korea, numerous *H*. *akashiwo* blooms have occurred in this area, and thus, DNA debris from these blooms may have still been present in the sediment samples.

A sieving and panning method could potentially reduce DNA debris, although because of the small size of *H*. *akashiwo* cysts (8–12 μm), such methods are very time-consuming due to the small sieve mesh size (>5 μm) required. Alternatively, we developed a simple dilution and heating method to remove DNA debris. DNA binds to silica through ion hydrogen bonds under high salt conditions in the presence of chaotropic agents such as KCl, NaCl, NaNO_3_, MgCl, and NaClO_4_ at low pH (pH ≤ 7) [39–41]. Marine sediments contain many silica materials, such as diatom debris, as well as high salt concentrations, a variety of chaotropic agents, and a lower pH than surface water [42]. Therefore, a large amount of extracellular DNA debris is likely bound to the silica components of marine sediments [23, 24]. Therefore, we used distilled water to dilute the chaotropic agents and heating at 75°C to break the hydrogen bonds between the DNA debris and silica material in the sediment. Although the removal of DNA debris with DW was not significant in our study, the treatment was slightly more effective than the control (Fig 4), most likely due to the fact that DNA binds to silica under high salt conditions. By contrast, heat treatment at 75°C had a significant effect on the removal of DNA debris from the sediment.

The mechanism of DNA debris removal in high temperature could be related with activity of nuclease presence in the sediment. However, according to Nielsen *et al*. [43], nuclease could be inactivated in sediment samples while nuclease has high activity in water samples. Moreover, 75°C condition is too high to activate nuclease because optimal temperature of nuclease activity is around 37°C [44]. Thus, we only could suppose that heating at 75°C might be sufficient to break the forces between the silica material and DNA due to hydrogen bonds are relatively weak.

It was possible that this DNA removal method could have resulted in cyst destruction. Although the effects of high temperatures on *H*. *akashiwo* cysts remain unknown, several studies have addressed the effects of high temperatures on other algal cysts [45–47]. Some species, including *Monomastix minuta* (Prasinophyceae), *Protosiphon* spp. (Chlorophyceae), and *Chlamydomonas* spp. (Chlorophyceae), have been shown to germinate after treatment for 1 h at 100°C or higher. The heat protection mechanisms of these algal cysts are not known, although it has been proposed that the thick cell walls of these cysts are a crucial factor.

*H*. *akashiwo* vegetative cells lack a cell wall, whereas cysts do have a cell wall [27]. The elemental composition of the *H*. *akashiwo* cyst wall is not known, but silicon is the predominant element (40%) comprising the *Chattonella marina* var. *antiqua* cyst wall, which like *H*. *akashiwo*, is a member of the Raphidophyceae class [48].

In this study, we confirmed that the *H*. *akashiwo* cyst cell wall can endure high temperature treatment. Furthermore, cell shape and nuclear integrity in the *H*. *akashiwo* cysts was not dramatically affected by treatment (Fig 5). We also confirmed that this DNA debris removal method was applicable to a variety of algal cysts other than *H*. *akashiwo*. Although we were unable to test all known cysts, all the cysts isolated in this study remained well preserved after treatment, regardless of size or type (Fig 6).

Therefore, treatments with DW and high temperatures do not appear to significantly affect algal cyst morphology, making these convenient and effective methods for removing DNA debris from sediment samples.

The DNA debris removal method and qPCR assay described here were successfully applied to field samples. We confirmed a significant correlation between qPCR- and microscopy-based results (*r*^2^ = 0.72, slope = 1.07, *p* < 0.001). Interestingly, results from samples without the DNA debris removal method also showed a significant relationship with the microscopic data, although the correlation coefficient value was lower than with the DNA debris removal method (*r*^2^ = 0.60, slope = 3.2, *p* < 0.001), indicating that the abundance of DNA debris in the sediment was similar to abundance of cysts. This finding suggests that the sedimentation of dead cells containing DNA debris is predominantly affected by local hydrodynamic factors, similar to the sedimentation of cysts themselves, suggesting that the amount of DNA debris may vary among sediment samples depending on the collection area. Another possibility is that considerable DNA debris is present on the surface of cysts, which is possible considering that cysts are covered with a viscous mucilage (Fig 5) that could absorb particles such as sediment and cell debris.

Although the qPCR quantification results without the DNA debris removal method still showed a significant relationship with the microscopic data, we observed considerable overestimation in these assays (S1 Fig). By contrast, qPCR results with the DNA debris removal method very closely reflected actual cyst number. Indeed, the gradient of the regression curve was nearly equal to one, indicating almost no over- or underestimation. Furthermore, these gradient values and high correlation coefficients were extremely reliable compared with a previous study involving qPCR and microscopic data [19].

DNA debris has been a significant hurdle to researchers trying to use qPCR-based assays to quantify cyst abundance, and as a result, this technique has been rarely used. In the present study, we developed a simple and effective DNA debris removal method and successfully performed qPCR using a cyst-based standard curve. We demonstrate that this qPCR assay can be used to accurately determine actual cyst numbers in the field. Therefore, this improved qPCR assay and DNA debris removal method should be a useful tool for further research on *H*. *akashiwo* and other phytoplankton cysts.

## Supporting Information

S1 Fig Comparison of cyst quantification results obtained from three methods. Quantification of *Heterosigma akashiwo* cysts in 18 sediment samples using a qPCR assay with specific real-time PCR probes after treatment in distilled water at 75°C (treatment) compared with no treatment (control). The white column shows results from the direct counting method (counting).
(TIF)
